# High vitamin K status is prospectively associated with decreased left ventricular mass in women: the Hoorn Study

**DOI:** 10.1186/s12937-021-00742-0

**Published:** 2021-10-19

**Authors:** Joline W. J. Beulens, Elisa Dal Canto, Coen D. A. Stehouwer, Roger J. M. W. Rennenberg, Petra J. M. Elders, Adriana Johanne van Ballegooijen

**Affiliations:** 1 Department of Epidemiology and Data Science, Amsterdam Public Health Research Institute, Amsterdam UMC–location VUmc, Amsterdam, The Netherlands; 2 Julius Center for Health Sciences and Primary Care, University Medical Center Utrecht, Utrecht, The Netherlands; 3 Medical Faculty, Amsterdam UMC, location VUmc, Van der Boechorststraat 7, 1081 BT Amsterdam, The Netherlands; 4 Department of General Practice, Amsterdam Public Health Research Institute, Amsterdam UMC–location VUmc, Amsterdam, The Netherlands; 5 Department of Internal Medicine and Cardiovascular Research Institute Maastricht (CARIM), Maastricht University Medical Center+, Maastricht, The Netherlands; 6 Department of Nephrology, Amsterdam Cardiovascular Sciences Research Institute, Amsterdam UMC–location VUmc, Amsterdam, The Netherlands

**Keywords:** Vitamin K status, Echocardiograph, Matrix gla protein, Vascular calcification

## Abstract

**Background:**

Vitamin K is associated with reduced cardiovascular disease risk such as heart failure, possibly by carboxylation of matrix-gla protein (MGP), a potent inhibitor of vascular calcification. The relationship of vitamin K intake or status with cardiac structure and function is largely unknown. Therefore this study aims to investigate the prospective association of vitamin K status and intake with echocardiographic measures.

**Methods:**

This study included 427 participants from the Hoorn Study, a population-based cohort. Vitamin K status was assessed at baseline by plasma desphospho-uncarboxylated MGP (dp-ucMGP) with higher concentrations reflecting lower vitamin K status. Vitamin K intake was assessed at baseline with a validated food-frequency questionnaire. Echocardiography was performed at baseline and after a mean follow-up time of 7.6, SD=±0.7 years. We used linear regression for the association of vitamin K status and intake with left ventricular ejection fraction (LVEF), left atrial volume index (LAVI) and left ventricular mass index (LVMI), adjusted for potential confounders.

**Results:**

The mean age was 66.8, SD=±6.1 years (51% were male). A high vitamin K status was prospectively associated with decreased LVMI (change from baseline to follow-up: -5.0, 95% CI: -10.5;0.4 g/m^2.7^) for the highest quartile compared to the lowest in women (*P*-interaction sex=0.07). No association was found in men. Vitamin K status was not associated with LVEF or LAVI. Vitamin K intake was not associated with any of the echocardiographic measures.

**Conclusions:**

This study showed a high vitamin K status being associated with decreased LVMI only in women, while intakes of vitamin K were not associated with any cardiac structure or function measures. These results extend previous findings for a role of vitamin K status to decrease heart failure risk.

**Supplementary Information:**

The online version contains supplementary material available at 10.1186/s12937-021-00742-0.

## Introduction

Vitamin K is a fat-soluble vitamin occurring in two biologically active forms; phylloquinone (vitamin K_1_) and menaquinones (vitamin K_2_) [1]. Vitamin K_1_ is mainly derived from green leafy vegetables, while vitamin K_2_ mainly occur in fermented animal products like cheese and meat. Vitamin K functions a co-factor for carboxylation (activation) of gla-proteins [2].

Matrix-gla protein (MGP) is a vitamin-K dependent protein and a potent inhibitor of vascular calcification [3]. Luo et al. showed that MGP knock-out mice died within 6-8 weeks due to massive calcification [3]. In humans, loss-of-function mutations in the gene coding for MGP result in the Keutel syndrome, which is characterized by abnormal calcification of cartilaginous tissues associated with malformations of skeletal tissues and arterial calcification [4]. Furthermore, animal and human intervention studies showed that vitamin K supplementation may reduce vascular calcification by carboxylating MGP [5–7].

Uncarboxylated MGP, as measured by desphospho-uncarboxylated MGP (dp-ucMGP) has been suggested as a marker for vitamin K status, with high concentrations indicating a low vitamin K status [5]. Although currently no single gold-standard marker is available to estimate vitamin K status, dp-ucMGP is considered the most suitable to assess vascular vitamin K status and measure vitamin K deficiency, as its concentrations correlate with long-term vitamin K intake [8, 9]. High concentrations of dp-ucMGP have been associated with increased vascular calcification [10] and risk of cardiovascular outcomes [11, 12], despite some conflicting results related to specific forms of cardiovascular disease (CVD) [13, 14]. Indeed, in a couple of studies the association between dp-ucMGP concentrations and coronary artery disease and stroke was absent [13] or appeared to be inverse [14]. Conversely, Dalmeijer et al. showed that the association between dp-ucMGP and CVD risk was mainly driven by heart failure (HF) and peripheral artery disease [11]. Use of vitamin K antagonists is also associated with increased vascular calcification in animal models [15, 16]. On the other hand, a higher vitamin K intake has been associated with a reduced risk of vascular calcification and CVD in prospective population-based cohorts [17].

Studies on the relationship of vitamin K intake or status with cardiac structure and function are, however, scarce. Objectively measured aspects of cardiac structure and function such as left ventricular mass index (LV, LVMI) or ejection fraction (LVEF) reflect the underlying hemodynamic alterations associated with HF, and echocardiography represents the gold standard for their assessment. In patients with HF, high dp-ucMGP concentrations were associated with the degree of LV systolic dysfunction [18] and with mortality [19]. Conversely, a high vitamin K intake has been associated with a reduced HF mortality [20]. Finally, three cross-sectional studies investigated the relation of vitamin K status with cardiovascular function, indicating significant associations between higher concentrations of dp-ucMGP and reduced LVEF [21], higher carotid-femoral pulse wave velocity [22] and depressed LV diastolic function [23]. However, prospective studies investigating the relationship between vitamin K status and intake with cardiac structure and function are currently lacking.

Therefore, the aim of this study is to investigate the association of vitamin K status and vitamin K intake with cardiac structure and function in a prospective, population-based cohort, the Hoorn Study.

## Materials/subjects and methods

### Study population

The Hoorn Study is a prospective observational cohort study in Dutch older adults initiated in 1989 with the aim to estimate the prevalence of pre-diabetes and diabetes in the general population. Adults living in Hoorn, in the region of the West-Friesland in The Netherlands, aged 50–75 years and able to provide consent were considered eligible. Among the eligible 11,500 participants, 3,553 were randomly selected from the municipal registry and 71.5% of those agreed to participate. After the exclusion of 56 participants of non-Western origin, 2,484 were included (age 61.7±6.7, 54.1% female) [24]. More detailed information on the research design, procedures and methods, has been published previously [24]. Participants of the Hoorn Study have been followed up via several visits. We included 831 participants who participated in extensive cardiovascular examinations in the year 2000-2001, considered as baseline. This group was oversampled for individuals with impaired glucose tolerance (IGT) and type 2 diabetes mellitus to enable investigation of effect modification by glucose tolerance status [24]. After 8 years of follow-up (2007-2009), a repeated echocardiogram was obtained in 438 participants (details about loss to follow up are displayed in Additional file 1).

Participants with unsatisfactory echocardiograms at follow-up, and with missing data on vitamin K status or intake for the respective analyses were excluded, resulting in 405 participants for analyses on vitamin K status and 427 participants for analyses on intake (Additional file 1). We did not have complete data on all three echocardiographic outcomes, which resulted in different analytic samples for each outcome measure with n=301 for LVEF, n=309 for LV mass index (LVMI) and of n=324 for left atrial volume index (LAVI) for vitamin K status and of n=312 for LVEF, n=324 for LVMI and n=335 for LAVI for vitamin K intake. All participants provided written informed consent and the local ethics committee of the VU University Medical Center approved the study.

### Vitamin K status

Study personnel collected morning blood samples in a 8-12 h fasted state. Citrated samples were stored at -80°C until assessment of biochemical measures. Vitamin K status was measured at baseline by plasma dp-ucMGP concentration. The analysis of dp-ucMGP were performed after the follow-up visits were completed, which was approximately 8-9 years after storage. Information on the stability of the biomarker during storage are not directly available from this study but from previous studies that used and tested the same procedure, showing a correlation of 0.67 for dp-ucMGP measurements taken over 5 years apart [10]. Dp-ucMGP concentration was measured by a sandwich ELISA with the capture of antibody directed against the nonphosphorylated MGP sequence 3 to 15 (mAb-dpMGP; VitaK BV, Maastricht, the Netherlands). High levels of dp-ucMGP reflect a low vitamin K status [5]. The interassay coefficient of variation was 9.9%.

### Vitamin K intake

At baseline, dietary intake was assessed using a self-administered validated food frequency questionnaire (FFQ) [25], allowing the estimation of the average daily intake of 178 foods over 77 main food categories in the year preceding enrolment. The 1993 Dutch food consumption table (NEVO) was used to calculate energy and nutrient intakes [26]. This table does not include information on vitamin K content of all food products. Therefore, the vitamin K_1_, short chain vitamin K_2_ and long chain vitamin K_2_ concentrations in a series of Dutch foods were assessed at the R&D Group VitaK, Maastricht University. These values were supplemented with earlier analyses by Schurgers and Vermeer and the U.S. food composition database [27]. Finally the vitamin K database was completed by estimating vitamin K1 and vitamin K_2_ content through recipes and ingredient information. The relative validity of the FFQ for vitamin K, with this database, has been assessed against 12 monthly 24-hour recalls among 121 men and women [27]. At the time dietary intake was assessed, the most commonly used dietary supplements in the Netherlands did not include vitamin K and vitamin K supplements were thus not questioned in the food-frequency questionnaire. Therefore, vitamin K intake reflected only dietary sources. The relative validity for energy adjusted vitamin K_1_, vitamin K_2_, short- and long- chain vitamin K_2_ intake were 0.34, 0.56, 0.32 and 0.66, respectively.

### Echocardiography

Experienced ultrasound analysts performed the echocardiographic imaging at baseline (2000/2001) and at follow-up (2007/2009), using the HP SONOS 5500 echocardiography system (2-4 MHz transducer, Andover, MA, USA) according to a standardized protocol consisting of two-dimensional, M-mode and pulsed wave Doppler assessments [28]. All echocardiograms were evaluated afterwards by a senior cardiologist.

Cardiac structure was measured by LVMI, which was calculated from linear dimensions by using the American Society of Echocardiography recommended formula and indexed to height^2.7^ [28]. LV systolic function was determined by LVEF (%), which was calculated using the modified Simpson’s rule [28]. As a marker of LV diastolic function the left atrial volume was measured at end-systole and indexed by body surface area resulting in LAVI (mL/m^2^) [29].

### Covariates

At baseline, participants were asked to fill out questionnaires about lifestyle factors, medical history (including family history of diabetes (yes/no), medication use (yes/no), disease history and diabetes-related complaints (yes/no), and smoking status (never/former/current smokers) [24]. Physical activity (h/day), including commuting activities, leisure time physical activity and occupational activities, was assessed by using the Short Questionnaire to Assess Health-enhancing physical activity (SQUASH), which has been previously validated [30]. Dietary intake of other nutrients than vitamin K was based on self-report using by the same validated FFQ [25]. Educational level was self-reported based on the highest ascertainment, and stratified according to three categories: 1) low (no/primary education); 2) middle (secondary education); 3 high (tertiary education). Information on prior and incident CVD was based on self-report in combination with medical records. CVD included myocardial infarction, angina pectoris, heart failure, stroke, transient ischemic attack and peripheral artery disease. In our morbidity and mortality registration, cardiovascular events were coded according to the International Classification of Diseases, Injuries and Causes of Death, ninth revision, including ICD-9 codes 390–459, and 798. Weight was divided by height squared to calculate body mass index (BMI, kg/m2). Blood pressure (mmHg) was measured twice at the left upper arm in a sitting position using an oscillometric device and averaged (Collin Press-Mate, BP-8800). Glucose tolerance status (NGT, IGT or diabetes) was determined using the results of an oral glucose tolerance test combined with the fasting glucose levels, according to the WHO 1999 criteria [31]. The estimated glomerular filtration rate (eGFR, mL/min) was calculated using the Chronic Kidney Disease Epidemiology Collaboration (CKD-EPI 2009) equation [32]. Plasma brain natriuretic peptide (BNP) was determined in spare frozen EDTA samples, which had been stored at –80°C for 4 years. BNP was determined in pmol/L (equivalent to 3.5 pg/mL) using an immunoradiometric assay kit (Shionoria, Osaka, Japan) with inter- and intra-assay variability coefficients in the relevant range <10% [33].

### Statistical analyses

All analyses were performed using SPSS Statistics, version 22.0 and R software version 3.1.1 (R-Foundation Statistical Computing). Variables were summarized as arithmetic mean and standard deviation for normally distributed data or as median with IQR range for data not normally distributed. Two-sided P-values of <0.05 were considered statistically significant. Vitamin K status and energy-adjusted intakes of all forms of vitamin K were categorized into quartiles. For vitamin K status the quartile with the highest dp-ucMGP concentrations and thus the lowest vitamin K status was used as the reference category. For quartiles of vitamin K intake, the lowest quartile was used as the reference category. Baseline characteristics were summarized by quartiles of vitamin K status and intake. Differences across quartiles of vitamin K status or intake were assessed by ANOVA for continuous measures and by a chi-square test for categorical variables.

Missing values for any covariates were present in 279 and 301 participants respectively for analyses on vitamin K status and intake, ranging from 0.2% for HbA1c to 2.9% for presence of CVD, and only BNP had 20.1% missing data. Multiple imputations were used combining ten iterations into one imputation model.

The longitudinal association of vitamin K status and intake with LVEF, LVMI and LAVI at follow-up were estimated using linear regression models adjusted for follow-up duration, glucose tolerance status and baseline value of the echocardiographic outcome. We additionally adjusted for confounders in a stepwise manner. Model 1 was adjusted for age and sex. Model 2 was additionally adjusted for BMI, smoking, physical activity, systolic blood pressure, presence of CVD, cholesterol, HbA1c and education. For vitamin K intake, we additionally adjusted for dietary factors associated with vitamin K intake: total energy intake, protein, saturated fat, fiber, calcium and vitamin C. All nutrients were modeled as continuous variables and adjusted for total energy intake by using the residual method [34]. The median level of vitamin K status or intake in each quartile was included as a continuous factor in the analysis to assess the P-trend over the categories.

We also included the quadratic term of continuous vitamin K status or intake along with the continuous term to assess evidence for deviation of a linear relationship. In case this quadratic term was significant, we used spline regression to explore the functional forms of the prospective associations between continuous vitamin K status or intake and echocardiographic measures.

Sex, glucose tolerance status and prior CVD were assessed as effect modifiers by including interaction terms in the fully adjusted models. Wald tests were used to assess whether the models with the interaction terms differed significantly from the initial models. A p-value <0.10 was considered statistically significant; in case interaction was present we stratified the results [35].

Sensitivity analyses were conducted by excluding cases with incident CVD during follow-up and by adjusting the final model for changes over time in smoking, physical activity and BMI. Finally, to investigate selection bias due to loss to follow-up, baseline characteristics of participants and dropouts were tested by an independent t-test for continuous variables and a chi-square test for categorical variables. Furthermore, a sensitivity analysis was performed using inverse-probability weighting for the longitudinal models.

## Results

### Baseline characteristics

Our study population for vitamin K status of 405 participants had a mean age of 66.8±6.1 years and 50.9% was male. At baseline, participants with a higher vitamin K status were younger, more frequently male, more frequently current smokers, had a lower systolic blood pressure and lower BMI than those with a low vitamin K status (Table 1). Among the 427 participants for vitamin K intake analyses, those with a high vitamin K intake were younger, less frequently male, lower educated, more physically active and had higher intakes of protein, fiber, vitamin C and calcium than those with a low vitamin K intake (Additional file 2). Those excluded in this study were older, had lower vitamin K status, higher prevalence of diabetes and prior CVD and slightly higher BMI and systolic blood pressure. Additionally, they showed a slightly worse cardiac structure and function, in particular a higher LVMI and a larger LAVI than those included in the analysis (Additional file 3).

**Table 1:** Baseline characteristics of 405 participants stratified by quartiles of vitamin K status

	Quartiles of dp-ucMGP (pmol/L)				
	Total population	Quartile 1	Quartile 2	Quartile 3	Quartile 4^a^
Dp-ucMGP (pmol/ xl) (min-max)	578±410 97-4608	258±70 97-366	432±41 366-507	597±56 508-697	1047±581 680-4608
n	405	102	103	102	98
Age (years)^d^	66.8±6.1	64.5±6.7	66.2±6.1	69.0±5.7	67.7±4.9
Male sex (%)^d^	50.9	56.9	59.2	47.1	39.8
High^b^ education (%)	22.0	22.5	27.2	19.2	19.8
Current smoking (%)	12.3	17.6	13.6	9.9	8.2
Physical activity (h/week)	22.5±17.0	22.2±15.7	24.0±19.8	21.5±17.3	22.1±14.8
Type 2 diabetes (%)	30.1	34.3	27.5	27.7	32.0
Previous CVD (%)	42.5	42.9	35.3	53.1	44.2
Systolic blood pressure (mmHg)^d^	139±394	134±20	139±17	142±19	143±20
Total cholesterol (mmol/L)	5.7±1.0	5.6±1.1	5.7±1.0	5.8±0.9	5.8±1.1
HDL cholesterol (mmol/L)	1.4±0.4	1.4±0.4	1.4±0.4	1.4±0.4	1.4±0.4
HbA1c (%)	6.0 ±0.8	5.9 ±0.7	6.0 ±0.8	6.0 ±0.7	6.1 ±0.8
BMI (kg/m^2^)^d^	27.3±3.5	26.3±3.2	27.4±3.9	27.4±3.2	28.3±3.3
eGFR (ml/min/1.73m^2^)^d^	63.7±10.2	68.1±10.3	64.1±9.5	61.8±10.5	60.6±8.7
*Echocardiographic measures*					
LVMI (g/m^2.7^)^d^	40.3±11.3	38.5±11.4	39.2±9.1	41.0±11.4	42.9±12.7
Ejection fraction (%)	62.0±8.0	61.4±7.3	62.6±7.6	60.8±8.8	63.3±8.0
LAVI (mL/m^2^)^d^	24.7±7.9	24.5±6.3	22.9±5.7	25.0±7.2	26.4±11.1
BNP (pg/mL)^d^	0.5 [0.2, 0.9]	0.4 [0.2, 0.7]	0.3 [0.2, 0.7]	0.7 [0.4, 1.1]	0.6 [0.3, 0.9]
*Dietary intake*					
Energy (kcal/day)	1980±508	1970±528	2056±512	2016±490	1875±489
Saturated fat (g/day)^c^	31.0±6.4	30.4±5.0	32.3±7.0	30.4±7.2	31.2±6.0
Protein (g/day)^c,d^	73.3±11.1	74.8±12.6	75.0±10.0	72.2±10.8	71.3±10.4
Fiber (g/day)^c,d^	24.3±4.7	25.3±4.7	24.0±4.5	24.6±4.4	23.2±4.9
Vitamin C (mg/day)^c^	107±42.1	109±43.8	105±36.4	109±41.4	107±46.9
Calcium (mg/day)^c^	1058±297	1065±333	1065±293	1073±281	1028±281
Alcohol (g/day)	7.2 [1.1-18.0]	6.9 [1.0-12.1]	8.2 [1.7-18.1]	7.5 [1.1-18.2]	5.9 [0.7-22.7]
Vitamin K_1_ (mg/day)^c^	185±75.8	189±76.9	192±69.8	184±83.1	172±72.2
Vitamin K_2_ (mg/day)^c^	35.4±13.6	37.7±16.4	35.6 ±12.2	33.2±13.1	35.0±12.1
Total vitamin K (mg/day)^c^	220±77.9	227±79.7	227±71.2	218±85.0	207±74.3
Short-chain vit K_2_ (mg/day)^c^	22.6±7.5	23.0±8.4	23.9±7.1	21.1±7.6	22.6±6.8
Long-chain vit K_2_ (mg/day)^c^	12.2±10.5	14.1±13.3	11.2 ±9.6	11.6 ±8.7	11.8±9.5

Values represent mean values and standard deviation, percentages or median (interquartile range)

^a^Quartile 4 indicates the highest dp-ucMGP concentration and therefore reflects the lowest vitamin K status

^b^High education indicates tertiary education

^c^Energy-adjusted intakes

^d^*p*<0.05 between quartiles of vitamin K status

### Vitamin K status

Mean vitamin K status was 578±410 pmol/L and showed a distribution somewhat skewed to the right. The mean follow-up time was 7.6 ±0.7 years.

**Fig. 1:**
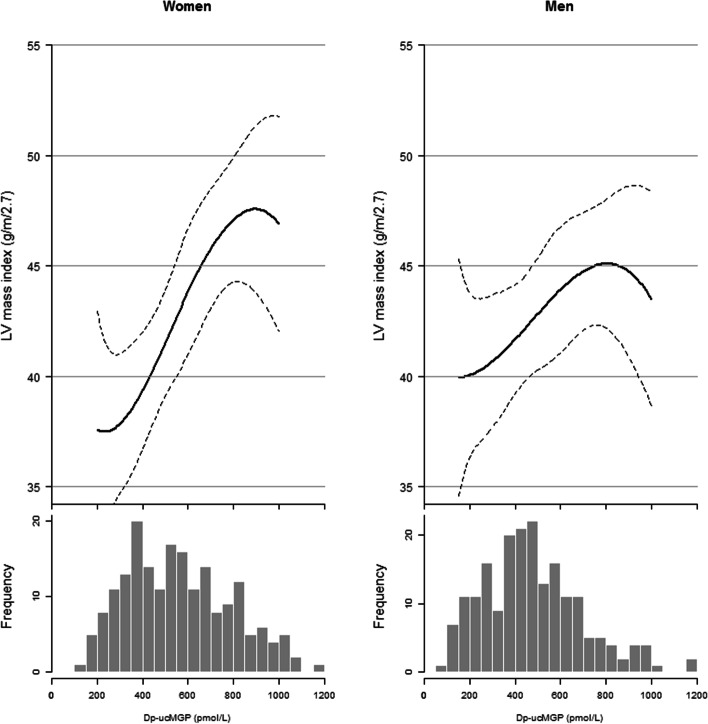
Continuous association of vitamin K status with left ventricular mass index stratified by sex among the inner 95% of concentrations. Women *N*=199; men *N*=206. Splines are adjusted for age, follow-up time, glucose status and baseline left ventricular mass index. Below each spline is the histogram depicted to illustrate the distribution of dp-ucMGP (pmol/L). High dp-ucMGP is indicative for low vitamin K status

In the linear regression models, including interaction terms for sex, glycemic status or previous CVD showed only evidence of interaction between vitamin K status and sex for LVMI (*P*=0.07) and results are therefore stratified by sex. Including a quadratic term in the model was statistically significant (P=0.018), suggesting evidence for a non-linear association. We observed a significant inverse relationship between vitamin K status and decreased LVMI in women for quartile 1 and 2 compared to quartile 4: -6.5(-11.2;-1.8) and -7.2(-11.9;-2.6) (g/m^2.7^) respectively, adjusted for model 1 (P-for trend<0.001). Further adjustment for model 2 slightly attenuated the association, and only quartile 2 was still significant: -6.8 (-11.8; -1.8). However the P-for trend remained strongly significant <0.001. In men, no significant association was observed. Evaluation of the continuous associations of vitamin K status with LVMI using splines revealed a similar pattern for men and women with some non-linearity in the tails (Figure 1). Vitamin K status was not associated with either LVEF or LAVI after follow-up with estimates of 1.1 (-2.4;4.5) (%) and -0.1 (-3.3;3.3) (mL/m^2^) for quartile 1 compared to quartile 4, respectively, adjusted for model 2 (Table 2).

**Table 2:** Prospective association between vitamin K status and echocardiographic measurements in 405 participants

	Quartiles of dp-ucMGP (pmol/L)				
	Quartile 1	Quartile 2	Quartile 3	Quartile 4^a^	***P***-trend
**LVMI (g/m**^**2.7**^**)**^b^					
*Men n=206*	40.9±9.2	40.7±10.4	43.6±13.3	45.2±11.7	
Model 1	-1.8 (-6.6; 3.0)	-0.7 (-5.4; 4.0)	2.1 (-2.9; 7.1)	Ref	0.353
Model 2	-1.3 (-6.4; 3.7)	-0.5 (-5.2; -4.1)	1.8 (-3.1; 6.8)	Ref	0.611
*Women n=199*	35.6±9.3	38.9±7.7	45.1±12.5	46.7±15.6	
Model 1	**-6.5 (-11.2;-1.8)**	**-7.2 (-11.9; -2.6)**	-0.3 (-4.8; 4.2)	Ref	**<0.001**
Model 2	-5.0 (-10.5; 0.4)	**-6.8 (-11.8; -1.8)**	0.4 (-2.9; 3.7)	Ref	**<0.001**
**LVEF (%)**^b^	53.4±9.2	53.3±9.8	52.0±11.2	52.3±10.2	
Model 1	1.3 (-1.9; 4.5)	1.0 (-2.1; 4.1)	1.3 (-1.9; 4.4)	Ref	0.48
Model 2	1.1 (-2.4; 4.5)	0.8 (-2.4; 3.9)	1.1 (-2.1; 4.3)	Ref	0.61
**LAVI (mL/m**^**2**^**)**^b^	24.0±9.4	25.2±10.1	26.0±10.6	28.0 ±18.0	
Model 1	-1.8 (-5.1; 1.4)	0.50 (-2.6; 3.6)	-0.8 (-4.0; 2.3)	Ref	0.43
Model 2	-0.1 (-3.3; 3.3)	0.9 (-2.2; 3.9)	-0.4 (-3.5; 2.6)	Ref	0.79

Model 1: Adjusted for baseline echo value (i.e. LVMI at follow-up adjusted for baseline LVMI), follow-up duration, age, sex and glycemic status

Model 2: Adjusted for model 1 and physical activity, smoking, BMI, systolic blood pressure, total cholesterol, HbA1c, education, prior CVD, BNP and eGFR

*Abbreviations*: *LVMI* left ventricle mass index, *LVEF* left ventricular ejection fraction, *LAVI* left atrium volume index

^a^Quartile 4 indicates the highest dp-ucMGP concentration and therefore reflects the lowest vitamin K status

^b^Mean echocardiographic measures at follow-up

P-for interaction vitamin K status and LVMI by sex=0.07

### Vitamin K intake

Mean total vitamin K intake was 220±78 mg/day (184 mg/day vitamin K_1_ and 35.5 mg/day vitamin K_2_). We did not observe significant associations between any of the forms of energy-adjusted vitamin K intake at baseline and LVMI at follow-up adjusted for CVD risk factors and dietary factors (Table 3). Similarly, for LVEF we observed no significant associations for any of the forms of vitamin K intake. Although we found some evidence of a non-linear association for total vitamin K (P=0.041) and vitamin K_1_ (P=0.059) with LVEF, the spline regression did not provide evidence for a significant association (Additional file 4).

**Table 3:** Prospective association between vitamin K intake and echocardiographic measurements in 427 participants

	Quartiles of vitamin K intake				
	Quartile 1^a^	Quartile 2	Quartile 3	Quartile 4	***P***-trend
n	106	107	107	107	
**LVMI (g/m**^**2.7**^**)**^b^	42.9±12.4	42.1 ±10.9	40.2±10.9	44.4±13.0	
Total vitamin K (mg/day)	Ref	-2.3 (-5.4; 0.8)	**-3.5 (-6.7; -0.3)**	-0.7 (-4.1; 2.6)	0.54
Vitamin K_1_	Ref	0.1 (-3.1; 3.1)	**-3.8 (-7.0; -0.6)**	0.3 (-2.9; 3.6)	0.57
Vitamin K_2_	Ref	-1.4 (-4.8; 2.1)	-0.6 (-4.2; 2.9)	-0.6 (-4.7; 3.5)	0.88
Long-chain	Ref	-0.1 (-3.4; 3.3)	-2.2 (-5.5; 1.1)	-0.9 (-5.0; 3.1)	0.32
Short-chain	Ref	-3.0 (-6.3; 0.3)	-1.7 (-5.4; 1.9)	-0.2 (-4.5; 4.1)	0.88
**LVEF (%)**^b^	52.2±9.8	53.4±9.5	54.3±11.1	51.2±10.4	
Total vitamin K (mg/day)	Ref	-0.3 (-3.3; 2.6)	-0.7 (-3.8; 2.3)	-1.6 (-4.4; 2.0)	0.29
Vitamin K_1_	Ref	1.3 (-1.7; 4.3)	1.2 (-2.1; 4.4)	-1.2 (-4.3; 2.1)	0.43
Vitamin K_2_	Ref	-1.3 (-4.6; 2.0)	-1.8 (-5.2; 1.5)	2.1 (-1.9; 6.1)	0.52
Long-chain	Ref	-1.1 (-4.3; 2.1)	-2.6 (-5.7; 0.5)	-1.2 (-4.9; 2.6)	0.31
Short-chain	Ref	-0.3 (-3.5; 2.9)	2.4 (-1.1; 5.9)	1.2 (-3.0; 5.5)	0.29
**LAVI (mL/m**^**2**^**)**^b^	25.3±11.7	26.0±11.7	26.6±14.0	26.6±12.1	
Total vitamin K (mg/day)	Ref	-0.2 (-3.1; 2.7)	-0.6 (-3.7; 2.5)	-1.6 (-4.7; 1.5)	0.29
Vitamin K_1_	Ref	0.2 (-2.7; 3.2)	-1.3 (-4.3; 1.8)	-1.1 (-4.2; 2.0)	0.33
Vitamin K_2_	Ref	1.8 (-1.3; 5.0)	1.8 (-1.5; 5.0)	1.5 (-2.3; 5.3)	0.46
Long-chain	Ref	2.7 (-0.2; 5.6)	0.8 (-2.2; 3.8)	2.1 (-1.4; 5.6)	0.48
Short-chain	Ref	3.1 (0.0; 6.2)	1.8 (-1.6; 5.2)	0.6 (-3.3; 4.6)	0.97

Adjusted for baseline echocardiographic value (i.e. LVMI at follow-up is adjusted for baseline LVMI), follow-up duration, age, sex, glycemic status, physical activity, smoking, BMI, systolic blood pressure, total cholesterol, HbA1c, education, presence of CVD, BNP, eGFR, energy intake and energy-adjusted intakes of protein, saturated fat, fiber, calcium and vitamin C

*Abbreviations*: *LVMI* left ventricle mass index, *LVEF* left ventricular ejection fraction, *LAVI* left atrium volume index

^a^Quartile 1 indicates the lowest vitamin K intake

^b^Mean echocardiographic measures at follow-up

In addition, we observed effect modification by presence of CVD for energy-adjusted vitamin K_2_ and long-chain vitamin K_2_ intake with LVEF (P-interaction<0.01), but no significant associations were observed when stratified for presence of CVD (Additional file 5). None of the forms of vitamin K intake at baseline were associated with changes in LAVI during follow-up.

### Sensitivity analyses

Excluding participants who developed CVD during follow-up (n=7) and adjusting for updated measurements of BMI, physical activity and smoking status at follow-up did not materially change our findings (Additional file 6). Adjusting for selection bias due to loss-to-follow-up using inverse probability weighting did not alter our findings as well (Additional file 6).

## Discussion

This study showed that a higher vitamin K status at baseline was associated with decreased LVMI after 7.6 years of follow-up, only among women. Vitamin K status was not associated with other cardiac measures. Vitamin K intake was not associated with cardiac structure and function.

This study is the first prospective study investigating the association between vitamin K status and cardiac structure and function measures. Our results are in line with earlier cross-sectional studies showing that a low vitamin K status was associated with reduced LV systolic function [21] or a higher prevalence of LV diastolic dysfunction [23]. However, in our study, a higher vitamin K status was associated with LVMI only among women and not among men, but such effect modification by sex was not investigated in earlier studies. We didn’t observe any association between vitamin K status and LVEF or LAVI. Nevertheless, the studies that found an associations between vitamin K status and LV systolic or diastolic function were performed in populations with different features than those of the present study, the participants being affected by valvular diseases, HF or cardiomyopathy. Despite this association between vitamin K status and LVMI, we could not detect any significant associations between any of the forms of vitamin K intake and any of the cardiac structure and function measures. This is inconsistent with the study by Eshak et al. showing that a higher vitamin K intake was associated with a 37% reduced risk of HF mortality after 19 years of follow-up among Japanese women [20]. The inconsistent findings between both studies could be due to the relatively low sample size of our study combined with the relatively low validity of the FFQ to accurately assess vitamin K intake.

The underlying mechanism of vitamin K status and intake, cardiac structure and function and risk of HF may be explained by improved arterial stiffness with increased vitamin K intake and status. Animal experiments indeed showed that antagonism of vitamin K leads to vascular calcification, in particular of the medial layer of the arteries [36]. Medial calcification leads to vascular stiffness [37, 38], which in turn is consistently associated with LV diastolic dysfunction [39]. In fact, vascular stiffness may increase the systolic load on the ventricles and decrease aortic pressure during diastole, thereby increasing pulse pressure [40]. Increased pulse pressure increases myocardial oxygen demand during systole and is associated with LV hypertrophy. These changes can lead to impaired relaxation and eventually LV diastolic dysfunction and HFpEF [41]. Indeed, earlier studies have shown vitamin K status to be associated with increased arterial stiffness [22, 42, 43]. One possible explanation of why in our study vitamin K status affected LV structure only in women, is that women, especially after menopause, seem to be more prone to develop LV hypertrophy in response to pressure overload compared to men. Consequently, the effect of vitamin K status on arterial stiffness and cardiac overload might manifest itself only in women [32].

Strengths of this study include its prospective design and detailed phenotyping of the participants, allowing to adjust for multiple confounders. However, certain limitations need to be addressed. First, the high loss to follow-up rates in this study reduced our sample size and might have biased the prospective associations. Although we observed similar results in our analyses after inverse probability weighting, we cannot exclude some selection bias by the high loss of follow-up rates from baseline to the second cardiovascular assessment. Second, because our study population included only people of West-European origin, the generalizability of our findings is limited to studies performed in people of Western descent. Third, for the assessment of vitamin K intake we relied on self-reported data. Although the FFQ was validated for the assessment of vitamin K intake, the relative validity in particular for the assessment of vitamin K1 and short-chain vitamin K2 was low. In combination with the relatively small sample size, this may have yielded the study insufficiently sensitive to detect associations with vitamin K intake. Finally, the definition of vitamin K status was based on a single baseline measurement of dp-ucMGP, which may only reflect short-term vitamin K status. Therefore these associations should be interpreted with caution.

## Conclusions

This study showed that a high vitamin K status is associated with decreased LVMI in women. Altogether these results further extend previous associations of vitamin K status with improved cardiac structure and function and a decreased risk of HF. An adequate vitamin K status, achieved via proper intake or possibly supplementation, might therefore be important to improve cardiac structure and function and prevent HF.

## Supplementary Information

**Additional file 1.** Flow chart of the Study Population selection.

**Additional file 2.** Baseline characteristics of 427 participants stratified by quartiles of vitamin K intake.

**Additional file 3.** Baseline characteristics of participants included and excluded for this study

**Additional file 4.** Prospective non-linear association between intake of energy-adjusted vitamin K intake with LVEF in 427 participants.

**Additional file 5.** Prospective association between vitamin K intake and left ventricular ejection fraction stratified by prior cardiovascular disease

**Additional file 6.** Sensitivity analyses for the prospective association between vitamin K status and echocardiographic measurements

## Data Availability

The datasets used and/or analyzed during the current study are available from the corresponding author on reasonable request.
