# Supplementary material for: High vitamin K status is prospectively associated with decreased left ventricular mass in women: the Hoorn Study
Source: Nutr J. 2021 Oct 19;20:85. doi: 10.1186/s12937-021-00742-0 (PMC8524956; doi:10.1186/s12937-021-00742-0)
Supplement: Supplementary file 1 — Additional file 1. Flow chart of the Study Population selection. [file 12937_2021_742_MOESM1_ESM.docx]

**Additional File 1:** Flow chart of the Study Population selection

Lost to follow-up for:

- Death (n=135);
- Health problems (n=54);
- Untraceable or moved out of the area (n=44);
- Not invited because of missing echocardiography at baseline (n=24);
- Other or unknown reasons (n=136)

Participants of the Hoorn study, baseline (2000-2001 (n = 831)

Participants of the Hoorn study, follow-up 2008-2009 (n = 438)

Excluded for:

- Missing data on Vitamin K status (n=29);
- Missing data on Vitamin K intake (n=7);
- Unsatisfactory echocardioraphy at follow-up (n=4)

**Study population**:

- Analyses on vitamin K status (n=405)
- Analyses on vitamin K intake (n=427)
