# Supplementary material for: High vitamin K status is prospectively associated with decreased left ventricular mass in women: the Hoorn Study
Source: Nutr J. 2021 Oct 19;20:85. doi: 10.1186/s12937-021-00742-0 (PMC8524956; doi:10.1186/s12937-021-00742-0)
Supplement: Supplementary file 2 — Additional file 2. Baseline characteristics of 427 participants stratified by quartiles of vitamin K intake. [file 12937_2021_742_MOESM2_ESM.docx]

**Additional File 2:** Baseline characteristics of 427 participants stratified by quartiles of vitamin K intake

|  | **Vitamin K intake (µg/day)** | | | | |
| --- | --- | --- | --- | --- | --- |
|  | **Total population** | **Quartile 1^1^** | **Quartile 2** | **Quartile 3** | **Quartile 4** |
| n | 427 | 106 | 107 | 107 | 107 |
| Vitamin K intake | 220±78 | 130±25 | 190 ±12 | 234±15 | 323±59 |
| (min-max) | 66-618 | 66-167 | 168-210 | 210-263 | 263-618 |
| Age (years) | 66.4±6.5 | 67.0±6.8 | 66.0±6.6 | 66.7±6.4 | 65.8±6.3 |
| Male sex (%) | 51.3 | 56.6 | 56.1 | 44.9 | 47.7 |
| High^2^ education (%) | 21.8 | 24.5 | 24.5 | 17.9 | 20.8 |
| Current smoking (%) | 11.9 | 11.3 | 11.2 | 12.1 | 13.1 |
| Physical activity (h/week) | 22.0±16.7 | 19.9±13.8 | 22.3±19.2 | 23.0±16.8 | 22.7±16.7 |
| Diabetes (%) | 34.2 | 35.9 | 32.1 | 34.3 | 35.8 |
| Previous CVD (%) | 42.2 | 39.2 | 36.9 | 45.3 | 51.4 |
| Systolic blood pressure (mmHg) | 139±19.3 | 139±18.1 | 139±19.0 | 141±20.3 | 137±20.0 |
| Total cholesterol (mmol/l) | 5.7±1.0 | 5.7±1.0 | 5.7±1.0 | 5.7±1.1 | 5.8±1.0 |
| HDL cholesterol (mmol/l) | 1.4±0.4 | 1.4±0.4 | 1.4±0.4 | 1.4±0.4 | 1.4±0.4 |
| HbA1c (%) | 6.0±0.8 | 6.0±0.8 | 6.0±0.7 | 6.1±0.8 | 6.1±0.8 |
| BMI (kg/m^2^) | 27.6±3.6 | 27.6±3.6 | 27.5±3.4 | 27.2±3.6 | 28.0±3.8 |
| Dp-ucMGP (pmol/l) | 579±413 | 636±408 | 524±292 | 565±412 | 594±510 |
| eGFR (ml/min/1.73m^2^) | 62.1±11.4 | 67.0±6.8 | 66.0±6.6 | 66.7±6.4 | 65.8±6.3 |
| *Echocardiographic measures* |  |  |  |  |  |
| LVMI (g/m^2.7^) | 40.5±11.3 | 39.5±10.5 | 41.1±12.1 | 40.0±10.9 | 41.2±11.8 |
| Ejection Fraction % | 62.0±7.9 | 61.4±7.7 | 63.3±6.9 | 62.2±7.8 | 61.2±9.2 |
| LAVI (mL/m^2^) | 24.7±7.9 | 23.8±7.1 | 24.6±8.3 | 24.7±7.7 | 25.5±7.8 |
| BNP (pg/ml) | 0.4 [0.2, 0.9] | 0.4 [0.2, 0.7] | 0.4 [0.2, 0.9] | 0.4 [0.2, 0.9] | 0.6 [0.2, 0.9] |
| *Dietary intake* |  |  |  |  |  |
| Energy (kcal/day) | 1986±515 | 1933±566 | 2011±500 | 1966±510 | 1974±488 |
| Saturated fat (g/day)^3^ | 30.9±6.4 | 31.3±7.9 | 30.9±5.5 | 30.7±5.1 | 30.8±6.8 |
| Protein (g/day)^3,4^ | 73.7±11.0 | 70.5±10.8 | 73.9±10.7 | 74.0±9.7 | 76.4±12.2 |
| Fiber (g/day)^3,4^ | 24.1±4.7 | 22.4±5.1 | 24.3±4.4 | 24.5±4.1 | 25.4±4.6 |
| Vitamin C (mg/day)^3,4^ | 108±42.1 | 94.8±41.0 | 106±43.9 | 112±37.6 | 118±42.7 |
| Calcium (mg/day)^3, 4^ | 1065±302 | 982±281 | 1081±302 | 1099±289 | 1099±324 |
| Alcohol (g/day) | 5.8 [0.4, 18.3] | 5.6 [1.0, 18.0] | 6.9 [1.0, 19.6] | 8.1 [1.6, 16.5] | 8.9 [1.2, 20.1] |
| Vitamin K_1_ (mg/day)^3,4^ | 184±75.8 | 99.3±24.4 | 156±17.1 | 197±17.6 | 284±61.2 |
| Vitamin K_2_ (mg/day)^3,4^ | 35.5±13.6 | 30.5±10.1 | 34.8±12.1 | 37.3±13.1 | 39.6±16.6 |
| Short chain vit K_2_ (mg/day)^3,4^ | 22.7±7.5 | 21.1±7.7 | 22.4±6.8 | 22.5±7.0 | 24.7±8.2 |
| Long-chain vit K_2_ (mg/day)^3,4^ | 12.3±10.4 | 9.0±7.6 | 11.7±9.6 | 14.1±10.7 | 14.4±12.5 |

^1^ Quartile 1 indicates the lowest vitamin K intake

^2^ High education indicates tertiary education

^3^ Energy-adjusted intakes

^4^ p<0.05 between quartiles of vitamin K intake

Values are mean**±**SD, percentages or median and interquartile range
