# Supplementary material for: High vitamin K status is prospectively associated with decreased left ventricular mass in women: the Hoorn Study
Source: Nutr J. 2021 Oct 19;20:85. doi: 10.1186/s12937-021-00742-0 (PMC8524956; doi:10.1186/s12937-021-00742-0)
Supplement: Supplementary file 3 — Additional file 3. Baseline characteristics of participants included and excluded for this study [file 12937_2021_742_MOESM3_ESM.docx]

**Additional File 3**: Baseline characteristics of participants included and excluded for this study

|  | **Included (N=405)** | **Excluded (N=426)** |
| --- | --- | --- |
| Dp-ucMGP (pmol/l)^4^ | 578±410 | 732±606 |
| Age (years)^4^ | 66.8±6.1 | 70.2±7.8 |
| Sex (% male) | 50.9 | 48.4 |
| High^1^ education (%)^4^ | 22.0 | 15.4 |
| Current smoking (%) | 12.3 | 17.8 |
| Physical activity (h/week)^4^ | 22.5±17.0 | 19.6±15.6 |
| Diabetes (%)^4^ | 30.1 | 52.6 |
| Previous CVD (%)^4^ | 42.5 | 52.9 |
| Systolic blood pressure (mmHg)^4^ | 139±394 | 145±21 |
| Total cholesterol (mmol/l) | 5.7±1.0 | 5.7±1.1 |
| HDL cholesterol (mmol/l) | 1.4±0.4 | 1.4±0.4 |
| HbA1c (%)^4^ | 6.0 ±0.8 | 6.2±0.8 |
| BMI (kg/m^2^)^4^ | 27.3±3.5 | 28.3±4.9 |
| eGFR (mL/min/1.73m^2^) | 63.7±10.2 | 61.6±9.8 |
| *Echocardiographic measures* |  |  |
| LVMI (g/m^2.7^)^4^ | 40.3±11.3 | 45.2±15.6 |
| Ejection Fraction (%)^4^ | 62.0±8.0 | 60.6±8.6 |
| LAVI (mL/m^2^)^4^ | 24.7±7.9 | 28.5±12.2 |
| BNP (pg/ml)^4^ | 0.7±0.8 | 1.3±2.1 |
| *Dietary intake* |  |  |
| Energy (kcal/day)^4^ | 1980±508 | 1873±538 |
| Saturated fat (g/day)^2^ | 31.0±6.4 | 30.6±5.8 |
| Protein (g/day)^2^ | 73.3±11.1 | 72.7±11.1 |
| Fiber (g/day)^2^ | 24.3±4.7 | 24.2±5.2 |
| Vitamin C (mg/day)^2^ | 107±42.1 | 106±46.5 |
| Calcium (mg/day)^2^ | 1058±297 | 1080±324 |
| Alcohol (g/day)^3^ | 7.2 (362.2) | 3.7 (19.5) |
| Vitamin K_1_ (mg/day)^2^ | 185±75.8 | 187±90.6 |
| Vitamin K_2_ (mg/day)^2^ | 35.4±13.6 | 33.6±13.7 |
| Total vitamin K (mg/day)^2^ | 220±77.9 | 221±93.0 |
| Short-chain vitamin K_2_ (mg/day)^2^ | 22.6±7.5 | 21.7±7.2 |
| Long-chain vitamin K_2_ (mg/day)^2^ | 12.2±10.5 | 11.4±10.3 |

Data are presented as mean±SD;

^1^ High education indicates tertiary education^; 2^ Energy adjusted intakes; ^3^ Median (interquartile range)

^4^ <0.05 between included and excluded participants
