# Supplementary figures and images for: High vitamin K status is prospectively associated with decreased left ventricular mass in women: the Hoorn Study
Source: Nutr J. 2021 Oct 19;20:85. doi: 10.1186/s12937-021-00742-0 (PMC8524956; doi:10.1186/s12937-021-00742-0)

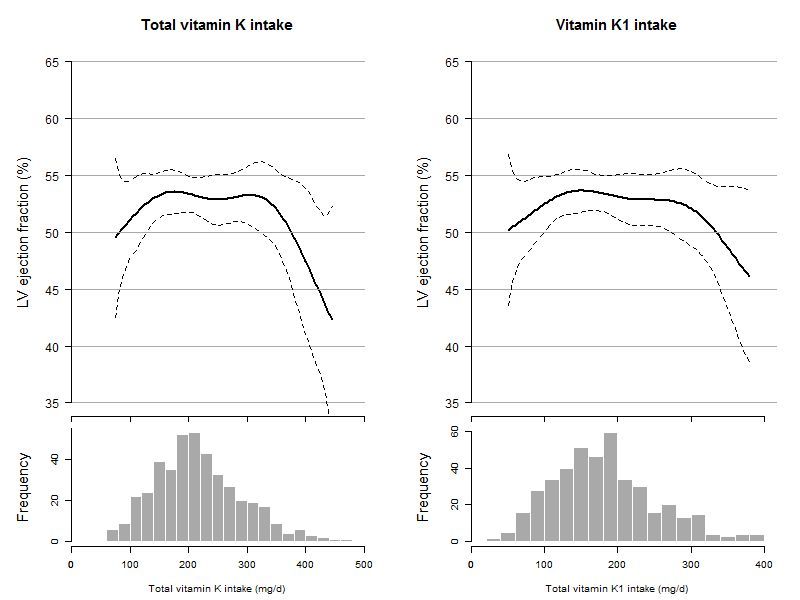

Supplement: Supplementary file 4 — Additional file 4. Prospective non-linear association between intake of energy-adjusted vitamin K intake with LVEF in 427 participants. [file 12937_2021_742_MOESM4_ESM.jpg]
