# Supplementary material for: High vitamin K status is prospectively associated with decreased left ventricular mass in women: the Hoorn Study
Source: Nutr J. 2021 Oct 19;20:85. doi: 10.1186/s12937-021-00742-0 (PMC8524956; doi:10.1186/s12937-021-00742-0)
Supplement: Supplementary file 5 — Additional file 5. Prospective association between vitamin K intake and left ventricular ejection fraction stratified by prior cardiovascular disease [file 12937_2021_742_MOESM5_ESM.docx]

**Additional File 5:** Prospective association between vitamin K intake and left ventricular ejection fraction stratified by prior cardiovascular disease

|  | | **Quartiles of vitamin K intake** | | | | |
| --- | --- | --- | --- | --- | --- | --- |
|  | **Quartile 1^1^** | | **Quartile 2** | **Quartile 3** | **Quartile 4** | **P-trend** |
| **LVEF (%)^2^** |  | |  |  |  |  |
| *No prior CVD (n=236)* | 62 | | 65 | 58 | 51 |  |
| Vitamin K_2_ | Ref | | -2.1 (-6.4; 2.2) | -2.2 (-6.6; 2.1) | 0.5 (-5.0; 6.1) | 0.92 |
| Long-chain | Ref | | 0.9 (-3.3; 5.1) | -3.2 (-7.1; 0.7) | -2.1 (-7.2; 2.9) | 0.12 |
| **LVEF (%)^2^** |  | |  |  |  |  |
| *Prior CVD (n=180)* | 40 | | 38 | 48 | 54 |  |
| Vitamin K_2_ | Ref | | -0.1 (-5.5 5.3) | -0.8 (-6.4; 4.8) | 3.2 (-2.7; 9.2) | 0.33 |
| Long-chain | Ref | | -1.7 (-6.7; 3.4) | -2.2 (-7.4; 3.0) | -2.8 (-6.1; 5.5) | 0.90 |

^1^ Quartile 1 indicates the lowest vitamin K intake.

^2^ Mean echocardiographic measures at follow-up

Abbreviations: LVEF left ventricular ejection fraction; CVD: cardiovascular disease

Adjusted for baseline echocardiographic value (i.e. LVEF at follow-up is adjusted for baseline LVEF), follow-up duration, age, sex, glycemic status, physical activity, smoking, BMI, systolic blood pressure, total cholesterol, HbA1c, education, presence of CVD, BNP, eGFR, energy intake and energy-adjusted intakes of protein, saturated fat, fiber, calcium and vitamin C.
