# Supplementary material for: High vitamin K status is prospectively associated with decreased left ventricular mass in women: the Hoorn Study
Source: Nutr J. 2021 Oct 19;20:85. doi: 10.1186/s12937-021-00742-0 (PMC8524956; doi:10.1186/s12937-021-00742-0)
Supplement: Supplementary file 6 — Additional file 6. Sensitivity analyses for the prospective association between vitamin K status and echocardiographic measurements [file 12937_2021_742_MOESM6_ESM.docx]

**Additional File 6:** Sensitivity analyses for the prospective association between vitamin K status and echocardiographic measurements

|  | **Quartiles of dp-ucMGP (pmol/L)­** | | | | |
| --- | --- | --- | --- | --- | --- |
|  | **Quartile 1** | **Quartile 2** | **Quartile 3** | **Quartile 4^1^** | **P-trend** |
| **LVMI (g/m^2.7^)^2^** |  |  |  |  |  |
| *Men n=206* | 40.9±9.2 | 40.7±10.4 | 43.6±13.3 | 45.2±11.7 |  |
| Base analysis | -1.3 (-6.4; 3.7) | -0.5 (-5.2; -4.1) | 1.8 (-3.1; 6.8) | Ref | 0.61 |
| Excluding CVD during follow-up | 0.1 (-5.2, 5.2) | 0.8 (-4.1, 5.6) | 3.0 (-2.1, 8.1) | Ref | 0.62 |
| Adjusted for updated confounders | -1.7 (-6.8, 3.5) | -0.9 (-5.8, 3.9) | 1.3 (-3.8, 6.4) | Ref | 0.33 |
| Inverse probability weighting | -1.3 (-6.4, 3.8) | -0.4 (-5.1, 4.3) | 2.0 (-3.1, 7.0) | Ref | 0.39 |
| *Women n=199* | 35.6±9.3 | 38.9±7.7 | 45.1±12.5 | 46.7±15.6 |  |
| Base analysis | -5.0 (-10.5; 0.4) | **-6.8 (-11.8; -1.8)** | 0.4 (-2.9; 3.7) | Ref | **<0.001** |
| Excluding CVD during follow-up | -4.9 (-10.3, 0.6) | **-6.8 (-11.7, -1.8)** | -0.2 (-5.0, 4.6) | Ref | **0.015** |
| Adjusted for updated confounders | -3.8 (-9.1, 1.4) | **-7.3 (-12.1, -2.5)** | 0.8 (-3.8, 5.4) | Ref | **0.018** |
| Inverse probability weighting | -5.3 (-10.7, 0.1) | **-7.5 (12.6, -2.5)** | -0.4 (-5.1, 4.4) | Ref | **0.009** |
| **LVEF (%)^2^** | 53.4±9.2 | 53.3±9.8 | 52.0±11.2 | 52.3±10.2 |  |
| Base analysis | 1.1 (-2.4; 4.5) | 0.8 (-2.4; 3.9) | 1.1 (-2.1; 4.3) | Ref | 0.61 |
| Excluding CVD during follow-up | 0.7 (-2.8, 4.2) | 0.1 (-3.1, 3.3) | 0.6 (-2.6, 3.8) | Ref | 0.79 |
| Adjusted for updated confounders | 1.0 (-2.4, 4.5) | 0.6 (-2.6, 3.8) | 1.1 (-2.2, 4.4) | Ref | 0.64 |
| Inverse probability weighting | 1.1 (-2.3, 4.0) | 0.8 (-2.3, 4.0) | 1.1 (-2.1, 4.3) | Ref | 0.58 |
| **LAVI (mL/m^2^)^2^** | 24.0±9.4 | 25.2±10.1 | 26.0±10.6 | 28.0 ±18.0 |  |
| Base analysis | -0.1 (-3.3; 3.3) | 0.9 (-2.2; 3.9) | -0.4 (-3.5; 2.6) | Ref | 0.79 |
| Excluding CVD during follow-up | 0.3 (-3.0, 3.6) | 1.2 (-1.9, 4.2) | -0.3 (-3.4, 2.8) | Ref | 0.64 |
| Adjusted for updated confounders | 0.1 (-3.1, 3.4) | 1.1 (-2.0, 4.1) | -0.2 (-3.3, 3.0) | Ref | 0.75 |
| Inverse probability weighting | -0.2 (-3.5, 3.0) | 0.6 (-2.4, 3.6) | -0.4 (-3.5, 2.6) | Ref | 0.92 |

^1^ Quartile 4 indicates the highest dp-ucMGP concentration and therefore reflects the lowest vitamin K status.

^2^ Mean echocardiographic measures at follow-up

Abbreviations: LVMI: left ventricle mass index; LVEF left ventricular ejection fraction; LAVI: left atrium volume index

Adjusted for baseline echocardiographic value (i.e. LVMI at follow-up is adjusted for baseline LVMI), follow-up duration, age, sex and glycemic status, physical activity, smoking, BMI, systolic blood pressure, total cholesterol, HbA1c, education, presence of CVD, BNP and eGFR
